# Microcalcification and Irregular Margins as Key Predictors of Thyroid Cancer: Integrated Analysis of EU-TIRADS, Bethesda, and Histopathology

**DOI:** 10.3390/medicina61122217

**Published:** 2025-12-16

**Authors:** Şebnem Çimen, Nazif Zeybek, Adile Begüm Bahçecioğlu, Kerim Bora Yılmaz, Neşe Ersöz Gülçelik, Mehmet Ali Gülçelik

**Affiliations:** 1 Department of General Surgery, Akyurt State Hospital, Ankara 06750, Turkey; 2 Department of General Surgery, Gülhane Training and Reseach Hospital, University of Health Sciences, Ankara 06010, Turkey; 3 Department of Endocrinology and Metabolism, Gülhane Training and Reseach Hospital, University of Health Sciences, Ankara 06010, Turkey; 4 Department of Surgical Oncology, Gülhane Training and Reseach Hospital, University of Health Sciences, Ankara 06010, Turkey

**Keywords:** thyroid nodule, EU-TIRADS, Bethesda System, ultrasonography, microcalcification, irregular margins, malignancy risk stratification, fine-needle aspiration biopsy, papillary thyroid carcinoma

## Abstract

*Background and Objectives:* Thyroid nodules are common, and distinguishing benign from malignant lesions is essential for clinical decision-making. While EU-TIRADS provides ultrasound-based risk stratification, fine-needle aspiration biopsy (FNAB) and the Bethesda System remain central diagnostic tools. This study aimed to compare the diagnostic performance of EU-TIRADS and Bethesda classifications and to identify ultrasonographic features independently associated with malignancy. *Materials and Methods:* This retrospective single-center study included 824 patients (1132 nodules) who underwent FNAB between August 2021 and June 2024. All ultrasound examinations and FNAB procedures were performed by the same endocrinologist. Sonographic features, EU-TIRADS categories, Bethesda classes, surgical indications, and histopathology were analyzed. Diagnostic accuracy was assessed using ROC curves, and multivariable logistic regression was applied to determine independent predictors of malignancy. *Results:* Among all nodules, 51.0% were EU-TIRADS 3, 28.6% were EU-TIRADS 4, and 19.2% were EU-TIRADS 5. Bethesda class II constituted 62.7% of FNAB results. Of the 289 surgically treated nodules, 53.3% were malignant. Malignant nodules were smaller, more often solitary and unilateral, and more frequently located in the upper pole (*p* < 0.05). Irregular margins (OR = 8.15, *p* < 0.001) and microcalcifications (OR = 10.01, *p* = 0.003) were independent predictors of malignancy. Taller-than-wide shape also showed significant association. ROC analyses demonstrated that EU-TIRADS (AUC = 0.808) and Bethesda (AUC = 0.869) were both significant predictors, with Bethesda showing higher specificity. Malignancy rates were 0% in EU-TIRADS II, 4.3% in III, 14.5% in IV, and 37.8% in V. *Conclusions:* EU-TIRADS is a practical and sensitive non-invasive tool for malignancy risk stratification; however, Bethesda classification remains superior in overall diagnostic accuracy. Microcalcification and irregular margins were the strongest ultrasonographic predictors of malignancy, while macrocalcification, parenchymal heterogeneity, and thyroiditis showed no significant association. These findings support the complementary roles of EU-TIRADS and FNAB and highlight key sonographic markers that enhance malignancy prediction in thyroid nodule evaluation.

## 1. Introduction

Thyroid nodules are very common, the majority of which are benign. Although the incidence of thyroid cancer has not increased, the prevalence of thyroid nodules has risen in recent years due to technological advancements and the widespread clinical use of ultrasonography. Distinguishing benign from malignant nodules is important for clinical approach and treatment planning. Nodules are evaluated through high-resolution ultrasonography and cytopathological examination [1].

Fine-needle aspiration biopsy (FNAB) remains the gold standard for cytopathological evaluation of thyroid nodules. FNAB prevents unnecessary thyroid surgeries and distinguishes patients with predominantly benign nodules from those with malignant ones. Before the routine use of FNAB, the malignancy rate detected in postoperative histopathological examinations was 14%, and with the introduction of FNAB, this success rate increased to nearly 50% [2]. To ensure a common language in cytopathological diagnosis, the Bethesda Scoring System was developed following the publication of American Thyroid Association (ATA) guidelines, and it has become the standard system [3]. The most recent revision in 2023 classified cytopathological samples into six categories: nondiagnostic, benign, atypia of undetermined significance, follicular neoplasm, suspicious for malignancy, and malignant [4].

The widespread use of ultrasonography and improved characterization of malignant features over time have raised the question of whether thyroid nodules can be radiologically differentiated as benign or malignant. For this purpose, several classification systems have been developed. Following the introduction of the BI-RADS (Breast Imaging Reporting and Data System) for breast lesions, Horvath et al. introduced the TI-RADS (Thyroid Imaging Reporting and Data System) for thyroid nodules in 2009, and in 2017 the American College of Radiology (ACR) released the ACR-TIRADS classification [5,6]. Subsequently, many institutions have published their own TIRADS systems.

The European Thyroid Association (ETA) also published its own classification, making the scoring system of ACR-TIRADS more applicable in clinical practice, and defined EU-TIRADS in 2017. EU-TIRADS categorizes nodules into five subgroups ranging from normal to highly suspicious for malignancy. During this evaluation, the nodule’s shape, margin characteristics, presence of microcalcifications, and echogenicity are assessed [7].

Because FNAB is an invasive procedure and requires time, cost, and workload for subsequent cytopathological examination—and to prevent unnecessary biopsies—performing benign–malignant differentiation by ultrasonography alone has become increasingly important. For this purpose, various classification systems have been extensively studied in the literature, and their specificity and sensitivity in identifying lesions have been evaluated [8,9,10].

In this study, we aimed to compare the diagnostic performance of EU-TIRADS scoring and Bethesda classification in differentiating benign and malignant nodules by examining the ultrasonographic features of patients who presented with thyroid nodules and subsequently underwent FNAB.

## 2. Materials and Methods

### 2.1. Collection of Patient Data

Adult patients over 18 years of age who underwent fine-needle aspiration biopsy (FNAB) for thyroid nodules at the University of Health Sciences Gülhane Training and Research Hospital between August 2021 and June 2024 were included. Among these, patients whose thyroid ultrasonography was reported by a single endocrinologist and who also underwent FNAB by the same endocrinologist were retrospectively selected. A LOGIQ P6 GE (GE Healthcare, Chicago, IL, USA) device was used during ultrasonography.

Patients with a history of thyroid surgery due to a thyroid nodule, those reoperated for recurrence, those who underwent completion thyroidectomy, those whose preoperative ultrasonography and/or FNAB were performed at an external center, and patients with concomitant parathyroid disease were excluded from the study.

FNAB was performed under ultrasonographic guidance using a 24–27-gauge needle (Becton Dickinson, Franklin Lakes, NJ, USA). After aspiration, the material was spread onto slides and fixed using the air-drying method. May Grünwald–Giemsa stain (Merck KGaA, Darmstadt, Germany) was applied, and the samples were examined under a light microscope (Olympus BX43, Olympus Corporation, Tokyo, Japan). The adequacy criterion required at least 6 follicular groups, each containing a minimum of 10 cells. For patients requiring repeat biopsy, the final FNAB result obtained before surgery was included in the study.

A total of 824 patients and 1132 nodules belonging to these patients were included.

Data collected from the hospital information system included patient age, sex, body mass index (BMI), thyroid hormonal status (hypothyroid, euthyroid, hyperthyroid), nodule size, number of nodules (single–multiple), nodule side (right–left), nodule location (upper, middle, lower), nodule distribution (unilateral–bilateral), ultrasonographic appearance of thyroiditis, parenchymal echogenicity (homogeneous, mildly heterogeneous, moderately heterogeneous, markedly heterogeneous), nodule composition (cystic, mixed, solid), nodule echogenicity (anechoic, hyperechoic, isoechoic, hypoechoic, markedly hypoechoic), nodule shape (ovoid, taller-than-wide), nodule margins (regular–irregular), presence of macrocalcifications, microcalcifications, linear microechogenicity, and comet-tail artifacts, EU-TIRADS category, Bethesda classification after FNAB, surgical status, histopathology (benign, low-risk neoplasm, malignant), and the specific histopathologic subgroup it belonged to (benign: thyroid follicular nodular disease, follicular thyroid adenoma, follicular thyroid adenoma with papillary architecture, oncocytic adenoma/low-risk neoplasm: NIFTP [noninvasive follicular thyroid neoplasm with papillary-like nuclear features], FT-UMP [follicular tumor of uncertain malignant potential], WD-UMP [well-differentiated tumor of uncertain malignant potential], hyalinizing trabecular tumor/malignant: follicular carcinoma, IEFV-PTC [invasive encapsulated follicular variant papillary thyroid carcinoma], oncocytic carcinoma, papillary carcinoma, DHGTC [differentiated high-grade thyroid carcinoma], PDTC [poorly differentiated thyroid carcinoma], anaplastic thyroid carcinoma, medullary thyroid carcinoma).

Initially, data were analyzed by comparing EU-TIRADS scores with cytopathological results based on the Bethesda classification. Subsequently, data from surgically treated patients were evaluated separately to assess the diagnostic performance of both EU-TIRADS and the Bethesda classification in predicting histopathological diagnosis.

### 2.2. Ethics Committee Approvals

Our study was designed as a single-center, retrospective study at the University of Health Sciences Gülhane Training and Research Hospital. Approval from the Educational Planning Committee (EPC) was obtained with the decision numbered “50687469.770.239806751” dated 3 April 2024 by the Educational Planning Committee of the Gülhane Training and Research Hospital Health Application and Research Center. Ethical approval was obtained from the Scientific Research Ethics Committee of the University of Health Sciences Gülhane with the decision numbered 2024-360 dated 28 June 2024 (code: 46418926). The study complies with the Declaration of Helsinki, Good Clinical Practice principles, and ethical rules for human research.

### 2.3. Statistical Analysis

Statistical analyses were performed using the SPSS version 22.0 software package. Descriptive statistics were expressed as number, percentage, mean ± standard deviation, and median. The normality of distribution for variables was evaluated using visual methods (histograms and probability plots) and analytical tests (Kolmogorov–Smirnov and Shapiro–Wilk tests). Although the sample size was large, several continuous variables, particularly nodule diameter and selected laboratory parameters, demonstrated non-normal distribution in Shapiro–Wilk testing; therefore, these variables were presented as median (IQR). For numerical variables with normal distribution, comparisons between two groups were made using the Independent Samples *t*-test. For numerical variables not showing normal distribution, the Mann–Whitney U test was used. For comparison of nominal variables, Chi-square analysis and Fisher’s Exact test were applied. Fisher’s Exact test was used when expected cell counts were <5. Agreement between the Bethesda and EU-TIRADS systems was assessed using Cohen’s kappa statistic. Kappa values were interpreted as follows: 0.00–0.20 insignificant, 0.21–0.40 low agreement, 0.41–0.60 moderate agreement, 0.61–0.80 substantial agreement, and 0.81–1.00 almost perfect agreement.

The predictive performance of EU-TIRADS and Bethesda classifications for malignant histopathology was evaluated using ROC analyses. ROC analyses were expressed with the area under the curve (AUC) along with 95% confidence intervals. After ROC analyses, optimal cutoff values demonstrating the best diagnostic performance were determined using the Youden Index. Sensitivity, specificity, positive predictive value (PPV), and negative predictive value (NPV) were calculated for these cutoff values.

Ultrasonographic features considered to be associated with malignant histopathology in univariate analyses were included in multivariate analyses. In the binary logistic regression analysis, malignant histopathology or high-risk Bethesda category was taken as the dependent variable, while markedly hypoechoic appearance, macrocalcification, microcalcification, taller-than-wide shape, and irregular margins were used as independent variables. Regression analysis results were expressed with odds ratios (OR) and 95% confidence intervals. A *p*-value < 0.05 was considered statistically significant.

## 3. Results

### 3.1. Descriptive Characteristics

A total of 1132 nodules from 824 patients were included in the study. The mean age of the patients was 49.4 ± 12.3 years (18–81 years). The female-to-male ratio was 4:1. The mean BMI of the patients was 28.3 ± 5.3 kg/m^2^. Most patients (73.8%) were euthyroid, while 14% were hyperthyroid and 12.3% were hypothyroid (Table 1).

The sonographic characteristics of the nodules were evaluated. The mean nodule size was 19.9 ± 12.6 mm. Most patients (85.2%) had more than one nodule. The location of the nodule was the right lobe in 47.1%, the left lobe in 43.5%, and the isthmus in 9.5%. Most nodules were located in the middle (44.1%) or lower (39.2%) pole. Thyroiditis was present in 73% of the cases. Parenchymal echogenicity was mildly heterogeneous in 40.5% of nodules, homogeneous in 41.8%, moderately heterogeneous in 12.5%, and markedly heterogeneous in 5.2%. The majority of nodules (87.3%) were solid or predominantly solid. Nodule echogenicity was isoechoic in 5.8%, hypoechoic in 39%, and markedly hypoechoic in 4.5% of nodules. Most nodules (95.3%) were ovoid-regular in shape. A “taller-than-wide” pattern was observed in 4.7% of nodules. Irregular margins were present in 14% of nodules. Macrocalcifications were present in 14.9%, microcalcifications in 4.2%, linear microechogenicity in 8%, and comet-tail artifacts in 1.8%. The sonographic characteristics of the nodules are shown in Table 2.

**Figure 1:**
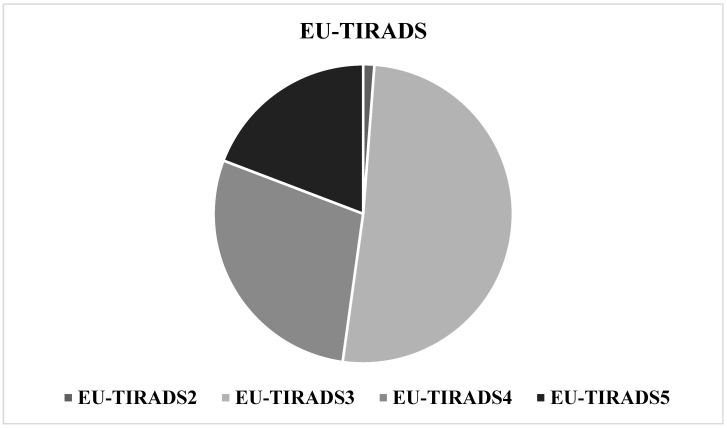
EU-TIRADS categories of thyroid nodules.

Based on the sonographic assessment, the distribution of thyroid nodules across the EU-TIRADS categories demonstrated a predominance of intermediate- and high-suspicion classes, with 1.2% categorized as EU-TIRADS 2 (n = 14), 51.0% as EU-TIRADS 3 (n = 577), 28.6% as EU-TIRADS 4 (n = 324), and 19.2% as EU-TIRADS 5 (n = 217) (Figure 1).

Based on the Bethesda classification of the materials obtained from FNAB, most nodules (62.7%) were classified as category II. The proportion of category V and VI nodules was 12.4%, while 7.7% were nondiagnostic (Category I). The Bethesda classification is shown in Table 3.

**Figure 2:**
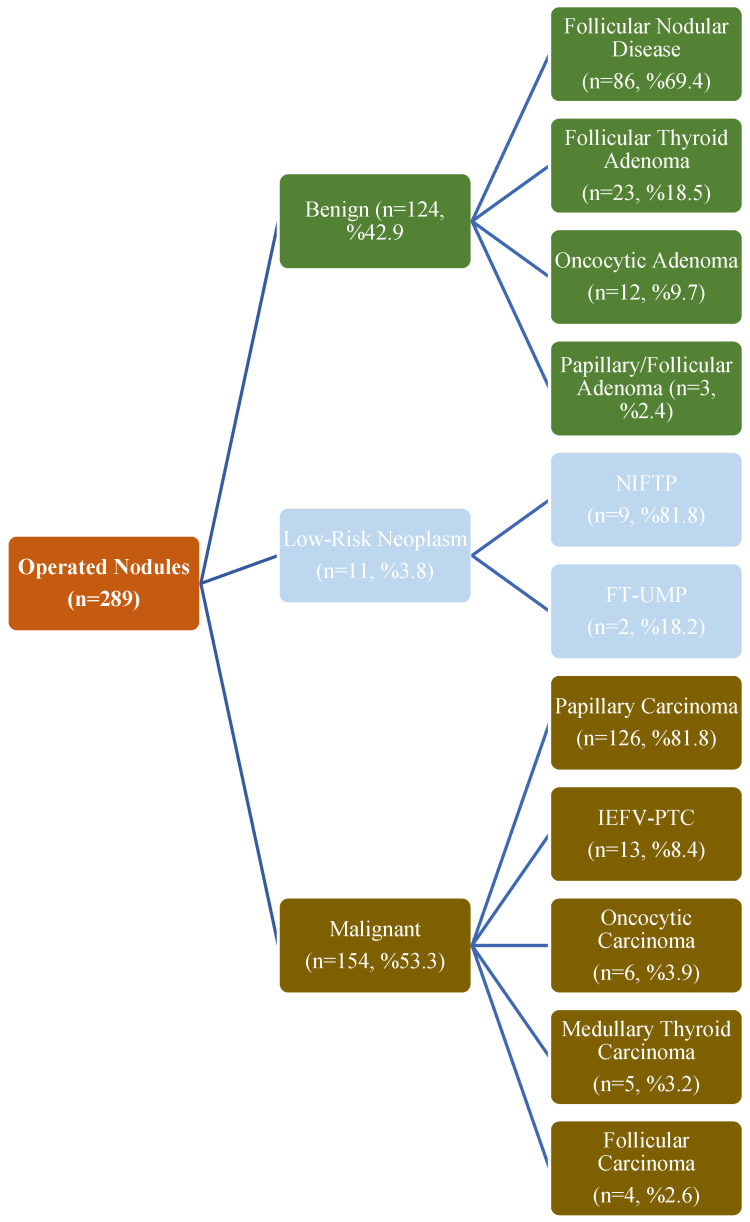
Histopathological evaluation results and final diagnoses of the operated nodules.

A total of 25.5% of the nodules (n = 289) were treated surgically. The most common surgical procedure was total thyroidectomy (70.9%). Lobectomy was performed in 13.9% of cases. Histopathological evaluation showed that 42.9% of the operated nodules were benign, 3.8% were low-risk neoplasms, and 53.3% were malignant. Among benign nodules, the most common types were follicular nodular disease (69.4%) and follicular thyroid adenoma (18.5%), whereas the most common malignant neoplasms were papillary thyroid carcinoma (81.8%) and IEFV-PTC (8.4%) (Figure 2).

### 3.2. Comparison of Benign and Malignant Nodules Based on Histopathology

The sonographic characteristics of benign and malignant nodules were compared. Malignant nodules were smaller in size compared with benign nodules (*p* < 0.001). The rate of solitary nodules (*p* < 0.001) and unilateral nodule distribution (*p* = 0.004) was higher in malignant nodules. There was a significant difference in the echogenicity patterns of benign and malignant nodules (*p* < 0.001). The “taller-than-wide” pattern was more frequently observed in malignant nodules (*p* = 0.001). Microcalcifications were also more common in malignant nodules (*p* < 0.001). The comparison of benign and malignant nodules is presented in Table 4.

### 3.3. Correlation Between the EU-TIRADS Classification and the Bethesda Classification

The concordance between the Bethesda and EU-TIRADS systems was evaluated using the kappa statistic. In the Bethesda system, categories IV, V, and VI were considered high risk, while categories II and III were considered low risk. In the EU-TIRADS classification, categories IV and V were considered high risk, whereas categories I, II, and III were considered low risk. The kappa analysis demonstrated a low but statistically significant agreement between the Bethesda and EU-TIRADS systems (kappa = 0.28, *p* < 0.001).

Among nodules classified as high risk in the Bethesda system, 62.3% were categorized as EU-TIRADS V, 30.2% as EU-TIRADS IV, and 7.5% as EU-TIRADS III. According to the Bethesda classification, the malignancy risk was 0% in EU-TIRADS II nodules, 2.2% in EU-TIRADS III nodules, 16% in EU-TIRADS IV nodules, and 49.7% in EU-TIRADS V nodules.

When histopathology was considered, none of the EU-TIRADS II nodules were malignant, whereas 4.3% of EU-TIRADS III nodules, 14.5% of EU-TIRADS IV nodules, and 37.8% of EU-TIRADS V nodules had malignant histopathology (Table 5).

Malignancy rates derived from histopathological outcomes demonstrated a stepwise increase across Bethesda categories, with malignancy observed in 8% of category I, 1.1% of category II, 13.6% of category III, 33.3% of category IV, 75.9% of category V, and 78.3% of category VI nodules, consistent with the progressive risk stratification defined by the Bethesda System.

### 3.4. Multivariate Analyses of Sonographic Features Associated with Malignancy Risk

Using malignant histopathology as the dependent variable, the effects of markedly hypoechoic appearance, the “taller-than-wide” pattern, irregular margins, and the presence of microcalcifications were analyzed among sonographic features. In univariate analyses, all sonographic characteristics except markedly hypoechoic appearance were associated with malignancy, whereas in multivariate analyses, irregular margins (*p* < 0.001) and the presence of microcalcifications (*p* = 0.003) were found to be independent predictors of malignancy (Table 6).

The predictive value of sonographic features for malignant histopathology was evaluated using ROC analyses. In the ROC analyses, the most discriminative features were irregular margins (AUC = 0.652), microcalcifications (AUC = 0.592), and the “taller-than-wide” pattern (AUC = 0.541). Markedly hypoechoic appearance alone was not a significant sonographic feature for malignant histopathology. For malignant histopathology, the sensitivity and specificity values were as follows: markedly hypoechoic appearance, 5.8% and 95.7%; taller-than-wide pattern, 11.6% and 96.4%; irregular margins, 40.2% and 90.1%; microcalcifications, 20.1% and 98.2% (Table 7).

### 3.5. Diagnostic Performance in Operated Nodules

The predictive value of the EU-TIRADS classification for malignant histopathology in operated nodules was evaluated using ROC analysis. The EU-TIRADS classification was found to be a significant standalone predictor for malignant histopathology (AUC = 0.808, *p* < 0.001).

**Figure 3:**
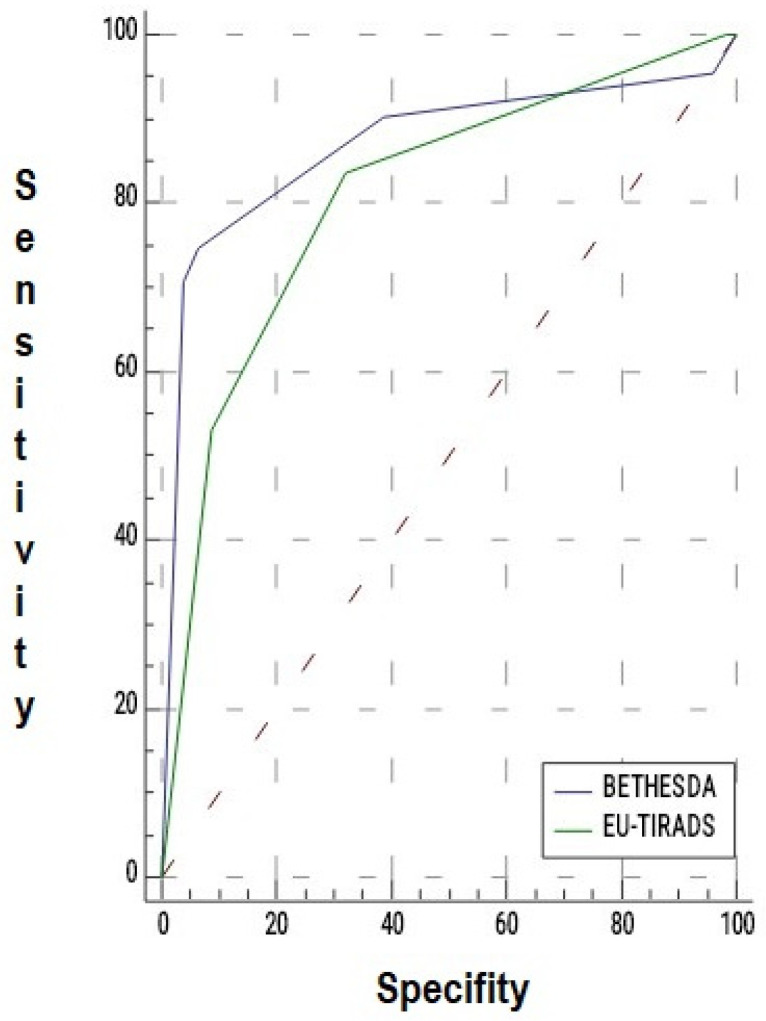
ROC curve of the Bethesda and EU-TIRADS classifications for malignant histopathology in operated nodules.

The predictive value of the Bethesda classification for malignant histopathology in operated nodules was also evaluated using ROC analysis. The Bethesda classification was determined to be a significant standalone predictor for malignant histopathology (AUC = 0.869, *p* < 0.001) (Table 8, Figure 3).

EU-TIRADS classification (>category III) demonstrated a sensitivity of 83.7% and a specificity of 67.7% for predicting malignant histopathology. In operated nodules, the Bethesda classification (>category III) showed a sensitivity of 74.6% and a specificity of 93.5% for malignant histopathology (Table 9).

## 4. Discussion

Thyroid nodules are commonly observed in the population, with an incidence reaching up to 24.8% [11]. Although most nodules follow a benign course, an increase in the incidence of thyroid cancer has been reported [3]. This may be attributed to technological advancements and the widespread use of ultrasonography, leading to the detection of more thyroid nodules. The detection rate of thyroid nodules by physical examination is around 5%, whereas with the use of ultrasonography, this rate increases to 20–76% [12,13]. The majority of thyroid nodules are asymptomatic and euthyroid [14]. Thyroid nodules are also more frequently observed in women. In one study, the prevalence of thyroid nodules was reported as 11.89% in women and 9.26% in men [15]. Additionally, previous studies have shown that increasing age and BMI are positively correlated with the prevalence of thyroid nodules and with the presence of multiple nodules [16,17]. Most nodules remain asymptomatic and are incidentally detected in euthyroid individuals. Elevated TSH levels, however, have been associated with malignancy in the literature [18].

In our study, the female-to-male ratio was found to be 4:1. A total of 73.8% of thyroid nodules were euthyroid, and 85.2% were detected as multiple nodules. Furthermore, hyperthyroidism appeared more frequent in benign than malignant nodules. However, no significant differences were observed between benign and malignant nodules with respect to age, sex, or BMI.

The relationship between nodule size and malignancy has been investigated in many studies, yet no consensus exists in the literature. While some studies have suggested that malignancy risk increases as nodule size increases [19,20,21], others have reported the opposite, demonstrating an association between smaller nodule size and malignancy [22,23,24,25]. In a study by Cavallo et al., the highest malignancy risk was observed in nodules measuring 2 cm or smaller [26]. Similarly, in our study, nodule size tended to be smaller in the malignant group.

In our study, the sonographic characteristics of the nodules were evaluated, and 85.2% of patients were found to have multiple nodules. Most nodules were identified as solid (87.3%). In a meta-analysis comparing solitary nodules with multinodular thyroids, malignancy was reported to be more frequent in solitary nodules [27]. Similarly, in our study, when solitary and multiple nodules were compared, the presence of a solitary nodule and, in multinodular cases, unilateral distribution showed a stronger association with malignancy.

Additionally, evaluation of nodule localization showed that upper pole location appeared more frequent among malignant nodules. Although previous studies have reported that nodules located in the isthmus are more likely to be malignant [28], in our study, no statistically significant association with malignancy was observed when isthmus, right lobe, and left lobe nodules were compared (*p* = 0.686).

When parenchymal echogenicity was evaluated, previous studies have demonstrated that the degree of parenchymal heterogeneity is not statistically meaningful in differentiating benign from malignant nodules [29,30]. Similarly, in our study, parenchymal heterogeneity categorized as homogeneous, mildly heterogeneous, moderately heterogeneous, and markedly heterogeneous showed no significant association with malignancy. Although some studies in malignant patients have reported a positive correlation between the presence of thyroiditis and malignancy, particularly papillary thyroid carcinoma [31,32], another study examining the general population did not find a statistically significant association between chronic thyroiditis and malignancy [33]. Consistent with this, our study also did not demonstrate a clear relationship between the presence of thyroiditis and malignancy.

In our study, the sonographic features of nodule shape, nodule margins, nodule echogenicity, and the presence of microcalcifications were significant indicators of histopathological malignancy. The presence or absence of macrocalcification was not associated with malignancy. Previous studies have identified hypoechogenicity as a marker of malignancy, and by evaluating varying degrees of hypoechogenicity, a positive correlation between increasing hypoechogenicity and malignancy has been demonstrated [34,35,36]. Similarly, irregular nodule margins have been shown to indicate malignancy with a specificity of 83.1%, and this feature has been incorporated into TIRADS systems [9,10,36]. Nodule shape and the presence of a “taller-than-wide” pattern have shown specificity values as high as 96.6% for malignancy in the literature [37,38]. Although some studies have reported an association between macrocalcification and malignancy [39,40], it has not been identified as an independent predictive factor for malignancy [40,41]. In contrast, the presence of microcalcifications has been described as an independent indicator of malignancy in the literature [34,42].

Our findings are largely consistent with those reported in previous studies evaluating sonographic predictors and TIRADS-based risk stratification, supporting the established role of microcalcifications, irregular margins, and hypoechogenic patterns in malignancy assessment. Although the novelty of individual associations may be limited, the strength of our study lies in its large sample size and the integrated evaluation of EU-TIRADS, Bethesda cytopathology, and final histopathology within the same cohort. This combined approach provides a more comprehensive perspective on diagnostic performance in real-world clinical practice and reinforces existing evidence with robust data. Importantly, our results support the continued use of EU-TIRADS as an effective triage tool for selecting nodules that require FNAB, while confirming that Bethesda cytopathology remains indispensable for final decision-making regarding surgery and clinical management.

In our cohort, malignancy rates increased stepwise across EU-TIRADS categories from II to V, in line with the ranges reported in the EU-TIRADS guidelines [7]. Similarly, malignancy risk increased across Bethesda categories I to VI and was broadly consistent with the 2023 Bethesda risk estimates, with some divergence in categories II and VI. We believe that the concordance between ultrasonographic assessment and the literature, and the partial discordance in cytopathological interpretation, may be attributed to the ultrasonographic evaluations being performed by a single physician, whereas cytopathological assessments were interpreted by different expert pathologists.

For statistical comparison, in our study, Bethesda categories IV, V, and VI were classified as high risk, while categories II and III were classified as low risk. In the EU-TIRADS classification, categories IV and V were defined as high risk, whereas categories I, II, and III were considered low risk. When the correlation between EU-TIRADS and the Bethesda cytopathological classification was evaluated, a statistically significant but low level of agreement was identified regarding malignancy (kappa = 0.28, *p* < 0.001).

In our study, when malignant histopathology was considered as the dependent variable and the sonographic features associated with malignancy in EU-TIRADS were analyzed, univariable analysis showed that all sonographic characteristics except marked hypoechogenicity were associated with malignancy. However, multivariable analysis confirmed that irregular margins and microcalcifications were the strongest independent ultrasound predictors of malignancy. Irregular margins increased the risk of malignancy eightfold, whereas the presence of microcalcifications increased the risk tenfold.

In the study by Na et al., 2000 nodules were evaluated, and within the hypoechoic group, irregular margins, a taller-than-wide pattern, and microcalcifications were identified as independent risk factors (*p* < 0.001) [41]. When the isoechoic and hyperechoic groups were analyzed separately, microcalcification and irregular margins were likewise determined to be independent risk factors (*p* = 0.002 and *p* = 0.015, respectively). In nodules with a partial cystic component, only microcalcification emerged as an independent risk factor (*p* = 0.006) [41]. Similarly, as reported by Kwak et al., microcalcification and irregular margins were more strongly associated with malignancy than hypoechogenicity or solid composition [43].

When the diagnostic performance of sonographic features was evaluated, marked hypoechogenicity alone was not discriminatory, whereas microcalcifications provided the highest specificity and positive predictive value, and irregular margins the highest sensitivity for malignancy. Similarly, in a study conducted in our country, microcalcifications also demonstrated the highest positive predictive value [44].

In the study by Ahn et al., which evaluated 1398 nodules, the most sensitive sonographic indicators were solid composition and irregular margins (89.3% and 84.4%, respectively), whereas microcalcification was found to be the most specific indicator of malignancy (98%). In the same study, PPV was highest for microcalcification (85.1%), exceeding all other nodule characteristics [45]. Likewise, in a study from Türkiye evaluating 1926 nodules, the PPV of microcalcification and irregular margins was higher than that of solid composition and hypoechogenicity (76% and 71.3% vs. 32.2% and 20.6%) [46].

In our study, because all sonographic assessments and the FNAB procedures for suspicious nodules were performed by the same physician, we believe that this consistency increased the reliability of our ultrasonographic evaluations.

In our study, EU-TIRADS showed good overall discriminatory ability for malignant histopathology, with high sensitivity but more modest specificity, comparable to previously reported performance metrics. In a previous study, the diagnostic ability of EU-TIRADS to detect malignant nodules was reported with a sensitivity of 96.6% and a specificity of 59.3% [47]. Another study, which included seven studies comprising 5672 nodules, reported sensitivity and specificity values of 83.5% and 84.3%, respectively, for EU-TIRADS 5 nodules [48]. In a large-scale meta-analysis evaluating 132 studies and multiple sonographic malignancy scoring systems, the sensitivity for EU-TIRADS categories III, IV, and V was found to be 93%, 93%, and 75%, respectively, and the corresponding specificity values were 17%, 51%, and 82% [49]. The same study also assessed ACR-TIRADS and C-TIRADS, reporting sensitivity values of 71% and 75% and specificity values of 87% and 87% for TIRADS 5 nodules. Additionally, computer-based deep learning approaches were evaluated and demonstrated a sensitivity of 81% and a specificity of 77% [49]. Overall, EU-TIRADS appears to outperform alternative systems in terms of sensitivity. In another study evaluating nodules Bethesda category III and above, EU-TIRADS showed a stronger correlation with Bethesda compared with ACR-TIRADS (*p* = 0.005 for ACR-TIRADS, *p* < 0.001 for EU-TIRADS) [50].

We also found that the Bethesda classification provided excellent discriminatory performance, with higher specificity than EU-TIRADS, in line with previously reported sensitivity and specificity ranges for FNAB-based systems [51,52,53,54]. When our results are compared with those of the EU-TIRADS system, it is evident that the Bethesda classification continues to demonstrate superior diagnostic performance and remains indispensable in clinical practice. These findings do not suggest a change in current surgical indications; however, ultrasonographic features such as microcalcifications and irregular margins may help refine FNAB selection in clinical practice, while Bethesda cytopathology remains essential before operative decision-making.

This study has several limitations. First, it was conducted in a single center and in a retrospective design. This inherently introduces selection bias in terms of patient inclusion and data quality. Although all ultrasonographic evaluations were performed by a single physician, which increases data consistency, it did not allow for the assessment of interobserver variability. Histopathological confirmation was available only for the nodules that underwent surgery; therefore, the true malignancy status of low-risk nodules that did not proceed to surgery could not be fully ascertained. The absence of molecular testing is another limitation, as such tests could have provided additional diagnostic information, particularly for nodules in the gray-zone categories. Considering all these factors, our findings should be interpreted with caution, and further large-scale, prospective, multicenter studies are warranted to validate and expand upon these results.

## 5. Conclusions

In this study, EU-TIRADS demonstrated good diagnostic utility for identifying suspicious thyroid nodules, while the Bethesda system remained superior for definitive malignancy prediction. Microcalcifications and irregular margins were the strongest independent sonographic predictors of malignancy, whereas macrocalcification, parenchymal heterogeneity, and thyroiditis showed no meaningful association. These findings highlight the complementary nature of EU-TIRADS and Bethesda in clinical practice: EU-TIRADS provides effective non-invasive risk stratification, whereas FNAB and cytopathology continue to be essential for accurate benign–malignant differentiation. Future prospective, multi-center studies incorporating advanced imaging and molecular testing may further enhance non-invasive diagnostic performance.

## Figures and Tables

**Table 1:** Baseline demographic and clinical characteristics of the patients.

Characteristic		Number (Percentage)
Age *	824	49.4 ± 12.3
Gender	824	
Female		661 (80.2)
Male		163 (19.8)
BMI (kg/m^2^) *	411	28.3 ± 5.3
Hormonal status	824	
Hypothyroid		101 (12.3)
Euthyroid		608 (73.8)
Hyperthyroid		115 (14.0)

BMI: body mass index. * BMI was available for 411 patients. Mean ± standard deviation is presented for continuous variables.

**Table 2:** Sonographic characteristics of thyroid nodules.

Characteristic (n = 1132)	Number (Percentage)
Nodule size (mm) *	19.9 ± 12.6
Number of nodules	
Single	168 (14.8)
Multiple	964 (85.2)
Nodule side	
Right	533 (47.1)
Left	492 (43.5)
Isthmus	107 (9.5)
Nodule location (n = 1027)	
Upper	171 (16.7)
Middle	453 (44.1)
Lower	403 (39.2)
Nodule distribution	
Unilateral	282 (24.9)
Bilateral	850 (75.1)
Presence of thyroiditis	826 (73.0)
Parenchymal echogenicity	
Mildly heterogeneous	458 (40.5)
Moderately heterogeneous	142 (12.5)
Markedly heterogeneous	59 (5.2)
Homogeneous	473 (41.8)
Nodule composition	
Cystic/predominantly cystic	15 (1.3)
Mixed	129 (11.4)
Solid/predominantly solid	988 (87.3)
Nodule echogenicity	
Anechoic	37 (3.3)
Hypoechoic	442 (39.0)
Isoechoic	598 (52.8)
Hyperechoic	4 (0.4)
Markedly hypoechoic	51 (4.5)
Nodule shape	
Ovoid–regular	1079 (95.3)
Taller than wide	53 (4.7)
Nodule margins	
Regular	974 (86.0)
Irregular	158 (14.0)
Macrocalcification	169 (14.9)
Microcalcification	48 (4.2)
Linear microechogenicity	91 (8.0)
Comet-tail artifact	20 (1.8)

* Reported as mean ± SD.

**Table 3:** Distribution of thyroid nodules according to the Bethesda classification.

	Bethesda (n = 1132)	Number (Percentage)
1	Nondiagnostic	87 (7.7)
2	Benign	710 (62.7)
3	AUS	176 (15.6)
4A	Suspicious for follicular neoplasm	13 (1.2)
4B	Suspicious for oncocytic neoplasm	5 (0.4)
5	Suspicious for malignancy	58 (5.1)
6A	Papillary carcinoma	78 (6.9)
6B	Medullary carcinoma	5 (0.4)

AUS: atypia of undetermined significance.

**Table 4:** Comparison of the sonographic characteristics of nodules based on histopathology.

Characteristic	Benign (n = 124)	Malignant (n = 154)	*p*-Value
	N (%)	N (%)	
Nodule size (cm) *	26.2 ± 15.7	16.7 ± 11.9	<0.001 ^†^
Number of nodules			<0.001
Single	10 (8.1)	39 (25.3)	
Multiple	114 (91.9)	115 (74.7)	
Nodule side			0.686
Right	58 (46.8)	80 (51.9)	
Left	52 (41.9)	59 (38.3)	
Isthmus	14 (11.3)	15 (9.7)	
Nodule location			0.047
Upper	19 (17.3)	36 (25.9)	
Middle	52 (47.3)	72 (51.8)	
Lower	39 (35.5)	31 (22.3)	
Nodule distribution			0.004
Unilateral	22 (17.7)	51 (33.1)	
Bilateral	102 (82.3)	103 (66.9)	
Presence of thyroiditis	95 (76.6)	109 (70.8)	0.274
Parenchymal echogenicity			0.872
Mildly heterogeneous	49 (39.5)	64 (41.6)	
Moderately heterogeneous	16 (12.9)	15 (9.7)	
Markedly heterogeneous	4 (3.2)	5 (3.2)	
Homogeneous	55 (44.4)	70 (45.5)	
Nodule composition			0.130
Cystic/predominantly cystic	2 (1.6)	0	
Mixed	16 (12.9)	13 (8.4)	
Solid/predominantly solid	106 (85.5)	141 (91.6)	
Nodule echogenicity			<0.001
Anechoic	4 (3.2)	4 (2.6)	
Hypoechoic	32 (25.8)	103 (66.9)	
Isoechoic	84 (67.7)	37 (24.0)	
Hyperechoic	0	1 (0.6)	
Markedly hypoechoic	4 (3.2)	9 (5.8)	
Nodule shape			0.001
Ovoid–regular	122 (98.4)	136 (88.3)	
Taller than wide	2 (1.6)	18 (11.7)	
Nodule margins			<0.001
Regular	116 (93.5)	92 (59.7)	
Irregular	8 (6.5)	62 (40.3)	
Macrocalcification	23 (18.5)	34 (22.1)	0.469
Microcalcification	2 (1.6)	31 (20.1)	<0.001
Linear microechogenicity	12 (9.7)	16 (10.4)	0.845
Comet-tail artifact	3 (2.4)	1 (0.6)	0.218

* Mean ± SD; ^†^ Independent samples *t*-test was used; Chi-square test was applied for all other analyses.

**Table 5:** Malignancy risks of nodules according to the EU-TIRADS classification.

	Bethesda		Bethesda	Histopathology
	Low Risk Bethesda II–III	High Risk Bethesda IV, V–VI	Malignancy Risk	Malignancy Rate *
	N (%)	N (%)	%	%
EU-TIRADS-II	7 (0.8)	0	0	0
EU-TIRADS-III	527 (59.5)	12 (7.5)	2.2	4.3
EU-TIRADS-IV	252 (28.4)	48 (30.2)	16.0	14.5
EU-TIRADS-V	100 (11.3)	99 (62.3)	49.7	37.8

* Based on final histopathology.

**Table 6:** Univariate and multivariate analyses of sonographic features for malignant histopathology.

	Univariate Analyses			Multivariate Analyses		
	OR	%95 CI	*p* Value	OR	%95 CI	*p* Value
Markedly hypoechoic appearance	1.38	0.65–2.90	0.391	0.41	0.09–1.84	0.245
Taller than wide	3.56	1.96–6.47	<0.001	4.70	0.97–22.83	0.055
Irregular margins	6.19	4.21–9.09	<0.001	8.15	3.36–19.76	<0.001
Microcalcification	14.24	7.66–26.50	<0.001	10.01	2.20–45.43	0.003

OR: Odds ratio; CI: Confidence interval.

**Table 7:** ROC analyses and diagnostic performance of sonographic features for malignant histopathology.

	AUC		%95 CI		*p* Value	
Markedly hypoechoic appearance	0.508		0.478–0.537		0.439	
Taller than wide	0.541		0.511–0.570		0.002	
Irregular margins	0.652		0.624–0.680		<0.001	
Microcalcification	0.592		0.563–0.621		<0.001	
	**Sensitivity** **(%)**	**Specificity** **(%)**		**PPV** **(%)**		**NPV** **(%)**
Markedly hypoechoic appearance	5.8	95.7		17.6		86.6
Taller than wide	11.6	96.4		34.0		87.4
Irregular margins	40.2	90.1		39.2		90.6
Microcalcification	20.1	98.2		64.6		88.7

AUC: area under the curve; CI: confidence interval; PPV: positive predictive value; NPV: negative predictive value.

**Table 8:** ROC analysis of EU-TIRADS and Bethesda classifications for malignant histopathology in operated nodules.

	AUC	%95 CI	*p* Value
EU-TIRADS	0.808	0.756–0.852	<0.001
Bethesda	0.869	0.823–0.906	<0.001

AUC: area under the curve; CI: confidence interval.

**Table 9:** Diagnostic performance of EU-TIRADS and Bethesda classifications for malignant histopathology in operated nodules.

	Sensitivity (%)	Specificity (%)	PPV (%)	NPV (%)
EU-TIRADS (>III)				
>II	100.0	1.6	55.8	100.0
>III	83.7	67.7	71.2	77.7
>IV	53.2	91.1	80.6	61.1
>V	0	100.0	-	44.6
Bethesda (>III)				
>I	95.4	4.0	55.3	41.7
>II	90.2	61.2	74.3	83.5
>III	74.6	93.5	93.5	74.8
>IV	70.7	95.9	95.6	72.6
>V	42.2	97.5	95.6	57.6

PPV: positive predictive value; NPV: negative predictive value.

## Data Availability

The data sets used and/or analyzed during the current study are available from the corresponding author on reasonable request due to confidentiality considerations.

## Abbreviations

The following abbreviations are used in this manuscript:

FNAB	fine-needle aspiration biopsy
BMI	body mass index
AUS	atypia of undetermined significance
IEFV-PTC	invasive encapsulated follicular variant papillary thyroid carcinoma
DHGTC	differentiated high-grade thyroid carcinoma
PDTC	poorly differentiated thyroid carcinoma
NIFTP	non-invasive follicular thyroid neoplasm with papillary-like nuclear features
FT-UMP	follicular tumor of uncertain malignant potential
WD-UMP	well-differentiated tumor of uncertain malignant potential
AUC	area under the curve
CI	confidence interval
PPV	positive predictive value
NPV	negative predictive value
OR	odds ratio
EU-TIRADS	European Thyroid Imaging Reporting and Data System
ACR-TIRADS	American College of Radiology Thyroid Imaging Reporting and Data System
C-TIRADS	Chinese Thyroid Imaging Reporting and Data System
ATA	American Thyroid Association
TSH	thyroid-stimulating hormone
SD	standard deviation
